# National Health Insurance Notes and Comments

**Published:** 1923-01

**Authors:** 


### NATIONAL HEALTH INSURANCE NOTES AND COMMENTS.

**A Step Towards A Better Understanding.**—We learn that the Insurance Acts Committee of the British Medical Association are inviting representatives of Approved Societies, Insurance Committees and workers in the field of national health insurance generally to meet them in public conference on some date in February. The object is, we understand, to bring about a pooling of ideas and, as far as possible, a common understanding as to practical ways in which experience has suggested to insurance workers that the system can be improved. This is all to the good, and although the subject of insurance doctors' rate of payment will be excluded from the scope of the conference, the fact of the parties coming together with a common object cannot fail to lead to an exchange of views which will be a step towards an amicable solution of the other vexed questions which will have to be faced later on in the year. We hope that the conference will be thoroughly representative of all parties, and wholly successful.

**An Amazing Article.**— Our contemporary "Health" has been badly let down. This paper is the organ of the Federation of Medical and Allied Services, and its expressed object is to assist the formation of public opinion on health questions. Several pages of its issue for December are devoted to an article written by an insured pe son, who is stated in the leading article for the month to have made a special study of the National Health Insurance Acts. The article is published, we gather, so that the public shall be fully enlightened on the working of the National Health Insurance system, with regard to which the journal states that the Federation have no predetermined attitude. The article gives one the impression of having been written by a person who has made a special study, not of the Insurance Acts, but of the art of juggling with figures. In one paragraph it is stated that it may be assumed that about one-third of the sum disbursed in benefits was expended in administration; in another that the administration was "responsible for three-fourths of every twenty shillings disbursed under the Act." In a later paragraph the statement is made that during the period from July, 1912-1919, the workers, in return for the payment of nearly £9,000,000, secured benefits to the value of only £1,501,375. Various other figures, all more or less bewildering, are dotted about the article, together with such extraordinarily inaccurate statements as these: (1) That a person who comes to London from the provinces is cut off from his panel doctor, and loses the benefit of the moneys he has paid before he can claim fresh ones; (2) that a domestic servant on leaving her employment and being unable to find a suitable situation for a month, loses the whole of the benefits to which she would have been entitled had she remained in her situation. Where a person who is vouched for as having studied the Insurance Acts can find authority for such statements, or what interest it is to present-day readers to learn what Mr. Handel Booth said in 1916, we are quite at a loss to imagine. We very much hope that before our contemporary publishes further articles on the subject it will take care to see that the statements made are reasonably accurate, in pursuance of its object "to present to the unprejudiced individual a true guide for his conduct towards present or future legislation on this matter."

**The Vaccination of Insured Persons.**—An official announcement at the time of the recent smallpox outbreak made it clear that the panel doctor must vaccinate insured persons on his list, that is, unless he is prepared to take the responsibility of advising that vaccination is unnecessary. The fact that the treatment could be obtained from a public vaccinator would not in itself justify an insurance practitioner in refusing to vaccinate one of his insured patients who desired him to perform the operation. The cost of the vaccine is chargeable to National Health Insurance funds. This is another reminder of the comprehensive nature of the panel doctor's contract. He must give to his insurance patients the same treatment, whether preventive or otherwise, that he would give to his private patients in any case in which in his view such treatment is necessary. It is not open to doctors to say that this view of their obligations is a new one, as long ago the Solicitor to the British Medical Association advised that vaccination was clearly within the insurance contract.

**The Lock-up Surgery.**— We hope that every Insurance Committee having within its area any of those populous districts in which doctors find it more congenial to reside at some distance away from their surgeries will make it their business to give full effect to the recent regulation which requires a doctor to satisfy the Committee that his arrangements are such as to enable his obligations to be properly carried out. If in exceptional cases lock-up surgeries are to be tolerated at all, very precise and clear arrangements as to the services of a deputy must be enforced so that an insured person may get the services of his doctor in an emergency without any delay. The doctor's lot is not an enviable one, but if his practice is carried on in uncongenial surroundings he must do it whole-heartedly or be prepared to make way for someone else.

**Insured Patients and Hospital Treatment.**—One of the complaints sometimes directed against the panel doctor is that he is too ready to hand over his responsibility to the nearest hospital. With a certain type of doctor we believe this complaint to be well founded. The obvious course is for the hospital to refuse definitely to give to an insured person treatment which is within the competence of a general practitioner, except, of course, in an emergency. This, of course, applies equally whether the application is for out-patient treatment or for admission to a free bed.

